# Reaction of Thiosulfate Dehydrogenase with a Substrate Mimic Induces Dissociation of the Cysteine Heme Ligand Giving Insights into the Mechanism of Oxidative Catalysis

**DOI:** 10.1021/jacs.2c06062

**Published:** 2022-09-29

**Authors:** Leon P. Jenner, Jason C. Crack, Julia M. Kurth, Zuzana Soldánová, Linda Brandt, Katarzyna P. Sokol, Erwin Reisner, Justin M. Bradley, Christiane Dahl, Myles R. Cheesman, Julea N. Butt

**Affiliations:** † Centre for Molecular and Structural Biochemistry, School of Chemistry and School of Biological Sciences, University of East Anglia, Norwich Research Park, NorwichNR4 7TJ, United Kingdom; ‡ Institut für Mikrobiologie & Biotechnologie, Friedrich Wilhelms Universität Bonn, D-53115Bonn, Germany; § Yusuf Hamied Department of Chemistry, University of Cambridge, Lensfield Road, CambridgeCB2 1EW, United Kingdom

## Abstract

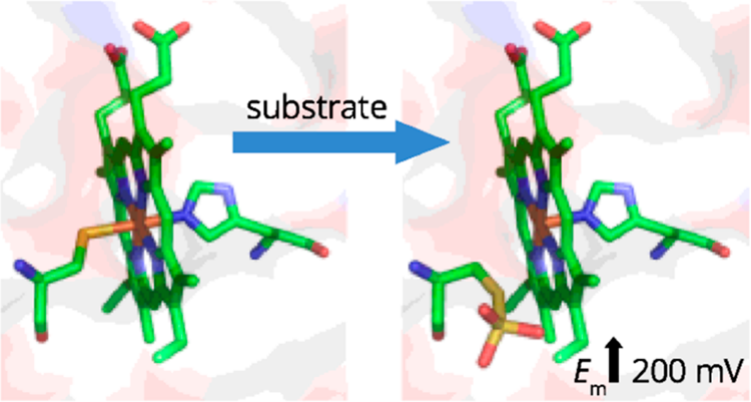

Thiosulfate dehydrogenases are bacterial cytochromes that contribute to the oxidation of inorganic sulfur. The active sites of these enzymes contain low-spin *c*-type heme with Cys^–^/His axial ligation. However, the reduction potentials of these hemes are several hundred mV more negative than that of the thiosulfate/tetrathionate couple (*E*_m_, +198 mV), making it difficult to rationalize the thiosulfate oxidizing capability. Here, we describe the reaction of *Campylobacter jejuni* thiosulfate dehydrogenase (TsdA) with sulfite, an analogue of thiosulfate. The reaction leads to stoichiometric conversion of the active site Cys to cysteinyl sulfonate (C_α_-CH_2_-S-SO_3_^–^) such that the protein exists in a form closely resembling a proposed intermediate in the pathway for thiosulfate oxidation that carries a cysteinyl thiosulfate (C_α_-CH_2_-S-SSO_3_^–^). The active site heme in the stable sulfonated protein displays an *E*_m_ approximately 200 mV more positive than the Cys^–^/His-ligated state. This can explain the thiosulfate oxidizing activity of the enzyme and allows us to propose a catalytic mechanism for thiosulfate oxidation. Substrate-driven release of the Cys heme ligand allows that side chain to provide the site of substrate binding and redox transformation; the neighboring heme then simply provides a site for electron relay to an appropriate partner. This chemistry is distinct from that displayed by the Cys-ligated hemes found in gas-sensing hemoproteins and in enzymes such as the cytochromes P450. Thus, a further class of thiolate-ligated hemes is proposed, as exemplified by the TsdA centers that have evolved to catalyze the controlled redox transformations of inorganic oxo anions of sulfur.

## Introduction

Hemes are arguably the most versatile cofactors in biology with roles in electron transfer, catalysis, and ligand binding for both transport and sensing, for example, refs (1)–^7^. These cofactors are characterized by a porphyrin ring that provides tetradentate ligation to iron via four nitrogen atoms in a square planar coordination. Where protein function requires the heme to bind substrates or exogenous ligands the iron typically has one axial ligand derived from the protein such that it is five-coordinate. Alternatively, the iron is six-coordinate with a labile group such as water or hydroxide trans from the protein-derived ligand. In contrast, the iron coordination sphere of hemes acting as electron-transfer sites is saturated by two protein-derived axial ligands, which results in a low-spin electronic configuration. The nature of these axial ligands is the prime determinant of the midpoint potential (*E*_m_) of the Fe(III)/Fe(II) couple.^8−12^ His/His axial coordination is by far the most common ligand set and supports an *E*_m_ in the range −350 to +300 mV (all potentials given vs S.H.E.), whereas examples of His/Met ligation appear restricted to sites that require elevated *E*_m_ values spanning the range +200 to +380 mV. Other six coordinate ligand sets defined by amino acid side chains have been reported and appear restricted to proteins with specialist function, for example, refs (6) and (13)–^19^.

Cysteine-derived thiolate axial ligation is common in heme-containing proteins that are five-coordinate or six-coordinate with one labile ligand. The best characterized of these proteins hold cysteinate-ligated *b*-type heme; two classes of such proteins have been identified^14^ based on spectroscopic characteristics that correlate with spin and oxidation state of the heme iron and that are in turn related to the lability of either the Cys ligand or the ligand trans to Cys. The type-1 proteins include the well-characterized O_2_ activating cytochromes P450 and nitric oxide (NO) synthases.^14^ In these enzymes, the cysteinate axial ligand supports catalysis by acting as a strong electron donor positioned trans to the site of small molecule binding and activation. The importance of the cysteinate is illustrated by comparing P450 enzymes to horseradish peroxidase (HRP), which has a histidine proximal ligand in place of cysteinate. The cysteinate ligand of cytochromes P450 results in catalytic intermediates, known as compounds I and II (Figure S1A), that are more powerful oxidants than the corresponding forms of HRP^20,21^ and may increase the efficiency of proton tunneling contributions in the abstraction, by compound I, of H^•^ from the substrate.^22,23^ Retention of cysteinate as a heme ligand to the ferric, ferrous, and ferryl states is therefore a requirement for type-1 protein function and is a defining feature of this class (Figure S1A). Because substrate activation oxidizes the heme iron, type-1 enzymes are located at the terminus of electron transport chains to receive electrons from NADPH and allow multiple turnovers.

The type-2^6,7,14^ heme-thiolate proteins are characterized by transitory cysteinate-heme binding for biological function (Figure S1B). Reduction of an intraprotein disulfide bond can release the cysteine thiolate required for His/Cys^–^ ligation of Fe(III)-heme, thereby allowing sensing of both cellular redox potential and heme levels. Further reduction to give the Fe(II)-heme can trigger dissociation of the Cys ligand (Figure S1B) to facilitate additional roles for these proteins as sensors of NO^24,25^ and CO.^26,27^ In principle, the type-2 proteins may contain two redox active centers, a disulfide bond and heme, but function does not involve continuous redox cycling of the heme iron. Therefore, in further contrast to the type-1 proteins, dedicated electron-transfer chains are not required for the biological function of the type-2 proteins.

Although omitted from the recent classification^14^ of Cys-ligated heme cofactors, such centers are also present in the active sites of bacterial thiosulfate dehydrogenases.^15−17,28,29^ These enzymes contribute to the redox cycling of inorganic sulfur for energy conservation. Found in phylogenetically diverse organisms, prominent examples are members of the SoxA and TsdA families. SoxA catalyzes the first step of the Sox thiosulfate oxidation pathway, which forms a disulfide bond between thiosulfate (^−^S–SO_3_^–^) and a cysteine residue on the SoxY carrier protein (Reaction 1).
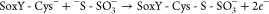
1

Through an analogous reaction, thiosulfate oxidation by TsdA enzymes involves the oxidative conjugation of two thiosulfate molecules to form tetrathionate (Reaction 2).
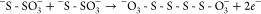
2

For both the SoxA and TsdA proteins, the active sites contain His/Cys^–^-ligated *c*-type heme covalently bound to the protein (Figure S1C). The resulting sites are low-spin with Fe(III)/(II) *E*_m_ values from −180 to −470 mV.^29−32^ These values fall in the more negative range of those displayed by His/His-ligated heme such that the requirement for a negative *E*_m_ is unlikely to be the sole reason for selecting the His/Cys^–^ ligand set in TsdA and SoxA proteins. Indeed, substitution of the Cys^–^ iron ligand in TsdA proteins produces inactive variants^17,33^ that retain heme at the active site due to its covalent attachment to the peptide. This is the case even for substitution of cysteine by histidine which increases the *E*_m_ by just 80 mV.^31^ Therefore, it seems likely that the Cys^–^ iron ligand assists catalysis in more ways than simply serving to modulate the thermodynamics of heme iron-based redox chemistry.

**Figure 1:**
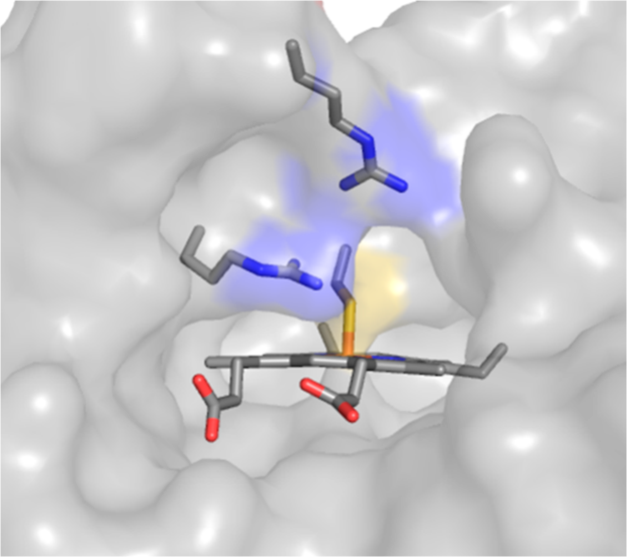
Cys^–^/His-ligated *c*-heme present in the active site of thiosulfate dehydrogenase, as illustrated for *A. vinosum* TsdA with Cys^123^, *c*-heme, Arg^109^, and Arg^119^ shown as sticks (PDB ID: 4WQ7).

Three further features of the thiosulfate dehydrogenases are striking with respect to their catalytic activity. First, a channel^15−17,28,29^ provides substrate access to the Cys-ligated side of the heme that is proposed as the catalytic pocket, for example, Figure 1. Second, the His/Cys^–^-ligated heme iron is six coordinate and therefore has no site available for the binding and activation of exogenous substrates. Third, the ability of these enzymes to perform thiosulfate oxidation is difficult to reconcile with their reported thermodynamic properties^30−32,34,35^ and as exemplified by TsdA from the food borne pathogen *Campylobacter jejuni* (*Cj*). A good indicator of the tendency for an oxidoreductase to act reversibly, or predominantly as either a reductase or an oxidase, is provided by the relative *E*_m_ values for the active site cofactor and the substrate/product couple.^36^ Given the thiosulfate/tetrathionate *E*_m_ of +198 mV,^35^ the His/Cys^–^-ligated active site heme of *Cj*TsdA with *E*_m_ −186 mV^31^ displays reasonable driving force for tetrathionate reduction but not for thiosulfate oxidation. This is difficult to reconcile with the ability of *Cj*TsdA to catalyze the thiosulfate oxidation to tetrathionate and the reverse reduction reaction at equivalent rates^33^ and with less than 10 mV overpotential.^35^

The thiosulfate anion (p*K*_a_ 0.6, 1.7) resembles tetrahedral sulfate (SO_4_^2–^) but with one oxygen replaced by sulfur. A mimic of thiosulfate is sulfite (SO_3_^2–^, p*K*_a_ 1.9, 7.2). Sulfite has trigonal pyramidal geometry, would be expected to bind to sites optimized to accommodate the three oxygen atoms of thiosulfate but lacks the additional sulfur atom required for the oxidative coupling catalyzed by TsdA. To investigate the catalytic mechanism of *Cj*TsdA, we have defined its reaction with sulfite. The active site of *Cj*TsdA contains *c*-type heme^31,33^ ligated by His^99^ and Cys^138^. In the product of the reaction with sulfite, Cys^138^ is present as a sulfonated form (C_α_-CH_2_-S-SO_3_^–^) and no longer a heme ligand. *E*_m_ for the Fe(III)/(II) couple of the corresponding heme is some 200 mV positive of that for the His/Cys^–^-ligated form. The sulfonated protein provides a stable mimic of a proposed intermediate in the pathway for thiosulfate oxidation that carries a cysteinyl thiosulfate (C_α_-CH_2_-S-SSO_3_^–^) such that our findings provide direct insights into the catalytic cycle of TsdA, and most likely SoxA, enzymes. Furthermore, they illustrate that while these enzymes share characteristics with both type-1 and type-2 systems, they cannot be placed into either category according to the definitions above. We therefore propose a further class of thiolate-ligated cytochromes, exemplified by those of TsdA and SoxA, which are optimized to catalyze the controlled redox transformations of inorganic oxo anions of sulfur.

## Results

*Cj*TsdA (hypothetical protein C8j_0815)^37^ is a diheme cytochrome^31,33,35^ predicted to fold as two cytochrome *c*-like domains based on sequence homology (Figure S1D) to the structurally defined^16,17^ TsdA from *Allochromatium vinosum* (*Av*). The *Cj*TsdA N-terminal domain contains the His^99^/Cys^138^-ligated active site heme (Heme 1) with *E*_m_ −186 mV.^31^ The C-terminal domain of this enzyme contains a His^207^/Met^255^-ligated *c*-type heme (Heme 2) with *E*_m_ of +172 mV^31^ and predicted to relay electrons between Heme 1 and cellular redox partners including periplasmic cytochrome *c*.^37^*Cj*TsdA was chosen for the present study because *Av*TsdA exhibits^17,31^ ligand switching and resultant complexity in Heme 2 redox properties that are not replicated in the *Cj* enzyme where the redox properties of the active site and electron-transfer hemes are more clearly delineated.^31^

**Figure 2:**
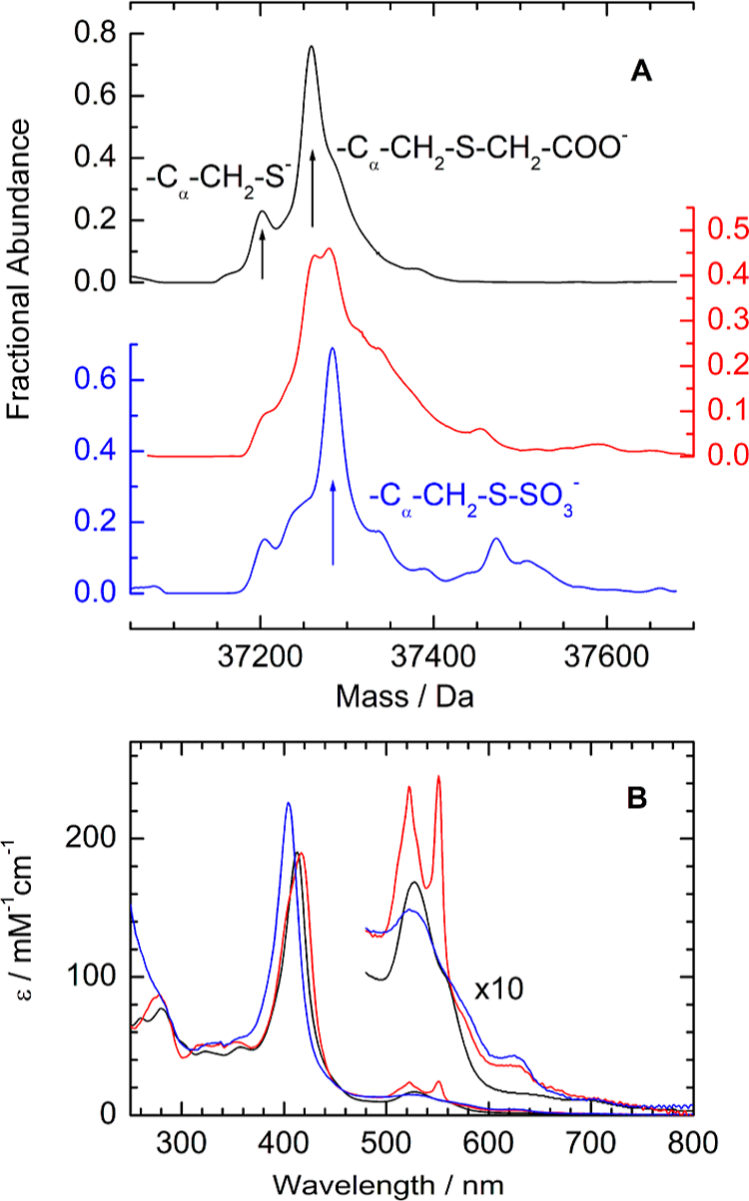
LCMS and electronic absorbance of di-Fe(III) *Cj*TsdA (black), after anaerobic incubation with 1.5 mM sulfite (red), then fully reoxidized with ferricyanide (blue). (A) Deconvoluted mass spectra after exposure to iodoacetate, see the text for details. Arrows indicate 37,202, 37,259, and 37,281 Da, the masses corresponding to *Cj*TsdA with cysteinate^138^ in unmodified, alkylated, and sulfonated forms, respectively, see the Supporting Information for details. (B) Electronic absorbance spectra. Samples (see Table S1 for the protein concentration) in anaerobic 50 mM HEPES, 50 mM NaCl, pH 7.

The *Cj*TsdA used in this study contained unmodified cysteinate^138^, and a mass of 37,202 Da was determined by LCMS (Figure S3). Treatment with iodoacetate^38,39^ to inhibit cysteinyl^138^ reactivity resulted in a dominant peak at 37,259 Da corresponding to the alkylated form (Figure 2A black). A small feature at 37,202 Da was assigned to a minor population, in which cysteinyl^138^ remained unreacted following iodoacetate treatment. After anaerobic incubation (45 min) of di-Fe(III) *Cj*TsdA with excess sulfite, followed by treatment with iodoacetate, LCMS revealed two poorly resolved species of comparable abundance (Figure 2A red). One species has the mass expected for the cysteinyl^138^ sulfonate adduct (37,281 Da), the other has the mass of alkylated *Cj*TsdA. After addition of an excess of the oxidant ferricyanide (*E*_m_, +420 mV) to the sulfite incubated protein, LCMS (Figure 2A blue), revealed almost exclusively the sulfonated form. Equivalent experiments using a variant protein with Cys^138^ replaced by His showed no evidence of protein sulfonation,^40^ and sulfite was found to inhibit both oxidative and reductive catalytic activities of *Cj*TsdA (Figure S6). Thus, sulfite is shown to bind to the active site of *Cj*TsdA and specifically through covalent attachment to Cys^138^.

**Figure 3:**
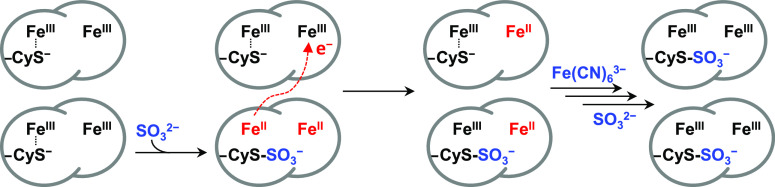
Reaction of di-Fe(III) *Cj*TsdA with sulfite. For clarity, only the heme irons and active site Cys^138^ are shown.

The observations above can be rationalized by the reaction scheme of Figure 3. Oxidative conjugation of sulfite to Cys^138^ releases two electrons, reducing both hemes of the originally di-Fe(III) protein. When interprotein electron transfer is faster than oxidative conjugation, and *E*_m_ Heme 2 > *E*_m_ Heme 1, electron redistribution according to *E*_m_ values would result in a semireduced sample, in which all Heme 2 is Fe(II) but only half the sample is sulfonated. Ferricyanide oxidation of Heme 2 restores di-Fe(III) protein and allows the remaining His/Cys^–^ Heme 1 to react with sulfite yielding a stoichiometrically sulfonated final product. The mechanism of Figure 3 is corroborated by spectroscopic and voltammetric studies described below, which also define the chemical and electrochemical properties of sulfonated *Cj*TsdA.

Changes in the electronic structure of *Cj*TsdA hemes on the reaction with sulfite were first investigated using electronic absorbance spectroscopy. Prior to sulfite addition, the absorbance spectrum (Figure 2B black) is typical of di-Fe(III) *Cj*TsdA^33^ containing only low-spin Fe(III) heme with a Soret band at 412 nm and broader weaker α/β bands at 500–570 nm. After the addition of sulfite, changes in the spectrum were noted over approximately 40 min after which time no further detectable changes occurred. The Soret band shifted to 417 nm (Figure 2B red), and sharp α/β-peaks at 551/522 nm report the formation of low-spin Fe(II) heme by sulfite-induced reduction. Importantly, the presence of high-spin Fe(III) heme is revealed by a small feature at ∼625 nm.^41^ Thus, the product of the sulfite-induced reduction is different to those elicited by the reductants, ascorbate (effective potential ≈+60 mV) and dithionite (*E*_m_ −500 mV) (Figure S5). These chemicals reduce, respectively, only Heme 2 or both hemes, but in all oxidation states the hemes remain low spin.^31,33,40^ Furthermore, the addition of ferricyanide to sulfite-treated *Cj*TsdA results in a spectrum (Figure 2B blue) that, while indicating Fe(III) heme only, is distinct from that of starting material in containing features from high-spin Fe(III), specifically the more intense and blue-shifted Soret band plus the persisting 625 nm feature.

**Figure 4:**
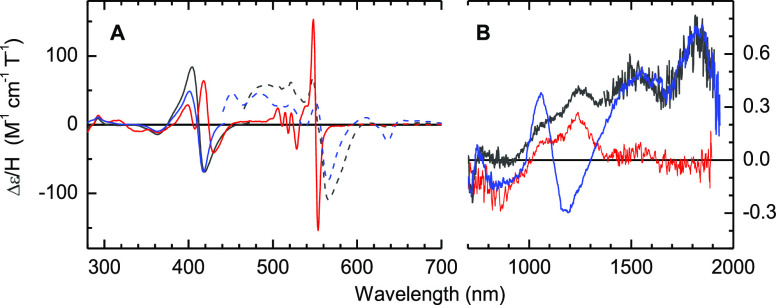
RT-MCD of di-Fe(III) *Cj*TsdA (black), after anaerobic incubation with 1.5 mM sulfite (red) followed by sufficient ferricyanide to fully reoxidize (blue). The dashed traces are expanded ×10. Samples (see Table S1 for protein concentrations) in anaerobic 50 mM HEPES, 50 mM NaCl, pH 7.

A more detailed characterization of the hemes in these samples was provided by magnetic circular dichroism (MCD) spectroscopy.^42^ All bands in the UV–visible MCD of the di-Fe(III) *Cj*TsdA starting material (Figure 4A black) arise from the two low-spin Fe(III) hemes, as previously reported.^31^ In the nIR region (Figure 4B black), bands at ∼1240 and 1825 nm are ligand-to-metal charge transfer transitions diagnostic of His/Cys^–^ and His/Met axial ligation at Hemes 1 and 2, respectively.^31^ Following anaerobic sulfite treatment (Figure 4A,B red), bisignate features at ∼423 and 551 nm are apparent and indicative of low-spin Fe(II) heme.^31^ The complete loss of the 1825 nm band reveals that this is due to reduction of the His/Met-ligated Heme 2.

Loss of intensity near 1240 nm implies a substantial decrease in the population of His/Cys^–^ Fe(III) Heme 1. A negative feature with λ_min_ = 636 nm indicates high-spin His/H_2_O-ligated Fe(III) heme.^42^ Addition of ferricyanide returns the protein to a homogeneous oxidized form (Figure 4A,B blue). The 1825 nm feature returns as Heme 2 is completely reoxidized with retention of His/Met ligation. The absence of the 1240 nm feature but emergence of CT transitions^42^ at 636 nm and 1110 nm show that Heme 1 is now high-spin Fe(III) with His/H_2_O ligation. Thus, cysteinyl sulfonate formed by sulfonation of Cys^138^ is no longer competent as a Heme 1 ligand and is replaced by water. While not characterized in this study, reduction of Heme 1 in the sulfonated protein is likely to trigger water dissociation and produce five-coordinate ferrous heme in behavior following that of the archetypical myglobin.^43,44^

**Figure 5:**
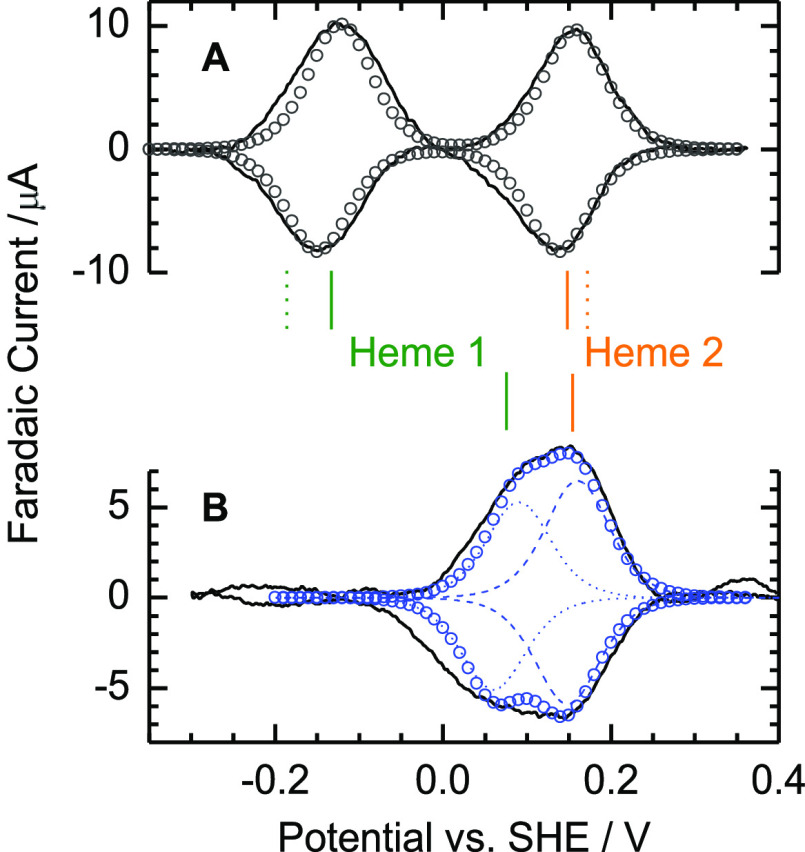
Representative protein film cyclic voltammograms for *Cj*TsdA before (A) and after (B) sulfite-conjugation of Cys^138^. Baseline subtracted current (lines). Circles show the sum of contributions from two *n* = 1 centers with *E*_m_ −134 and +145 mV (A) and *E*_m_, +75 and +154 mV (B) with individual contributions shown as dashed lines for the latter. These *E*_m_ values are also shown by the solid vertical lines for each heme. Dotted vertical lines show *E*_m_ values previously reported for *Cj*TsdA.^31^ Scan rate is 10 mV s^–1^ in anaerobic 50 mM HEPES, 50 mM NaCl, pH 7.

Having established a method to prepare homogeneous *Cj*TsdA samples with sulfonated cysteinyl^138^, the redox properties of that form were compared to those of protein with His/Cys^–^-ligated Heme 1 using protein film voltammetry. The cyclic voltammogram (Figure 5A) of the latter contains two, well-separated pairs of peaks describing reversible reduction of His/Cys^–^-ligated Heme 1 (*E*_m_, −134 ± 10 mV) and His/Met-ligated Heme 2 (*E*_m_, +145 ± 10 mV) as expected. The small difference between *E*_m_ values reported here and those reported previously^31^ is most likely due to heterogeneity of the covalent modification of Cys^138^ in the protein studied previously and not present in the starting material used for the studies reported here, see the Supporting Information for details. In contrast, cyclic voltammetry of the sulfonated protein shows redox activity only between approx. −50 and +250 mV (Figure 5B). The oxidative and reductive peaks are broader than expected for a single *n* = 1 process. Their features are well-described by overlapping contributions from two *n* = 1 processes from approximately equal populations of centers with *E*_m_, +75 and +154 mV (±20 mV). The higher value is assigned to His/Met Heme 2 as the MCD data (Figure 4) showed that the site is preferentially reduced in the sulfonated protein. The His/H_2_O-ligated Heme 1 is assigned an *E*_m_, +75 mV. Thus, replacing the Heme 1 Cys^138^ ligand with water has raised *E*_m_ by more than 200 mV with relatively little impact on the properties of Heme 2. LCMS of samples before voltammetry and recovered from electrodes after the experiments confirmed little change in the status of Cys^138^ during measurements.

## Discussion

Sulfite is an analogue of the thiosulfate substrate of *Cj*TsdA and an inhibitor of this enzyme. Here, we discuss the nature of the reaction between *Cj*TsdA and sulfite, as revealed by the presented data. We then explain how those data offer a mechanistic rationale for thiosulfate oxidation by TsdA and SoxA enzymes and compare the properties of the Cys-ligated heme in these enzymes to those of the previously defined^14^ type-1 and type-2 centers.

In this work, accurate mass determination by LCMS demonstrated that Cys^138^ is required for the modification of *Cj*TsdA induced by treatment with sulfite, and that an electron sink such as ferricyanide is essential for this process to reach completion. MCD of protein following this treatment showed that Cys^138^ is no longer a ligand to Heme 1, and protein film voltammetry revealed that this results in a significant change in the *E*_m_ value. Correlation of *E*_m_ values with heme ligation and oxidation state deduced from MCD demonstrated that the *E*_m_ of Heme 2 is unaffected by sulfite exposure while that of Heme 1 is increased by approximately 200 mV due to the dissociation of Cys^138^ induced by this substrate analogue. The observed mass increase on incubation of *Cj*TsdA with sulfite is that predicted for sulfonation, and it is logical to conclude that this is the consequence of covalent attachment of sulfite to Cys^138^ in a process that liberates two electrons transiently located on Hemes 1 and 2 (Figure 3). Indeed, this reaction mimics that described previously^45^ for free cysteine with sulfite in the presence of cupric ions
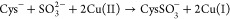
When the conjugation occurs in *Cj*TsdA, the electron acceptors critical for the reaction to proceed are provided by ferric Hemes 1 and 2.

A previous study has described the products of reducing one or both hemes in *Cj*TsdA by poising with the chemical reductants ascorbate or dithionite.^31^ It is significant that neither reaction triggers dissociation of Cys^138^ from the iron of Heme 1. While sulfite is produced by the oxidation of dithionite,^46^ we note that the corresponding reduction of *Cj*TsdA is almost instantaneous, whereas the spectral changes induced by chemical treatment with sulfite as described here require tens of minutes to reach completion. We postulate that rapid reduction of the *Cj*TsdA hemes by dithionite precludes sulfite modification of Cys^138^ as the protein is incapable of accommodating the additional electrons that would be liberated during oxidative conjugation. Thus, our results indicate that dissociation of Cys^138^ from Heme 1 in the presence of the substrate mimic sulfite is not simply a consequence of heme reduction.

Studies of Cys-heme ligation in proteins and models have illustrated a critical role for the Cys protonation state in the lability of this ligand.^47−49^ The Fe(III)-heme metalloporphyrin unit, with a +1 charge, favors thiolate over thiol ligation with the few examples of thiol-ligated ferric heme^47^ associated with highly electron-rich systems. Thus, the cysteinate ligation of Fe(III) Heme 1 in *Cj*TsdA is expected. Heme reduction increases the electron density on the iron, which in turn increases the effective p*K*_a_ of the coordinated thiol. This typically leads to ligand protonation and, although ferrous thiol coordination is possible, dissociation of the cysteine thiol is frequently observed as exemplified^14^ by type-2 cofactors (Figure S1B). The behavior^50^ of rat liver H-450 suggests that Cys ligated to the heme iron has a p*K*_a_ of <5 for the Fe(III) state and approximately 7 for the Fe(II) state. The corresponding values for Cys^138^ in *Cj*TsdA will reflect specific features in the environment of that residue. Sequence alignment (Figure S1D) to the structurally resolved TsdA enzymes from *A. vinosum*(16,17) and *Marichromatium purpuratum*(28) predicts that the Cys-ligated face of *Cj*TsdA lies in an Arg-rich pocket (Figure 1). The Arg side chains will contribute positive charge to that pocket such that Cys^138^ may well have a lower p*K*_a_ for both heme oxidation states than that for the corresponding states of H-450.

We infer that such considerations are relevant to the ligand dissociation triggered by sulfite prior to heme reduction in *Cj*TsdA. Our impression is that the high positive charge density of this pocket results in a sufficiently low p*K*_a_ of Cys^138^, even upon reduction of the heme iron, to prevent protonation and subsequent ligand dissociation unless charge compensation upon binding of sulfite results in an elevated p*K*_a_ and therefore protonation followed by cysteine thiol dissociation.

**Figure 6:**
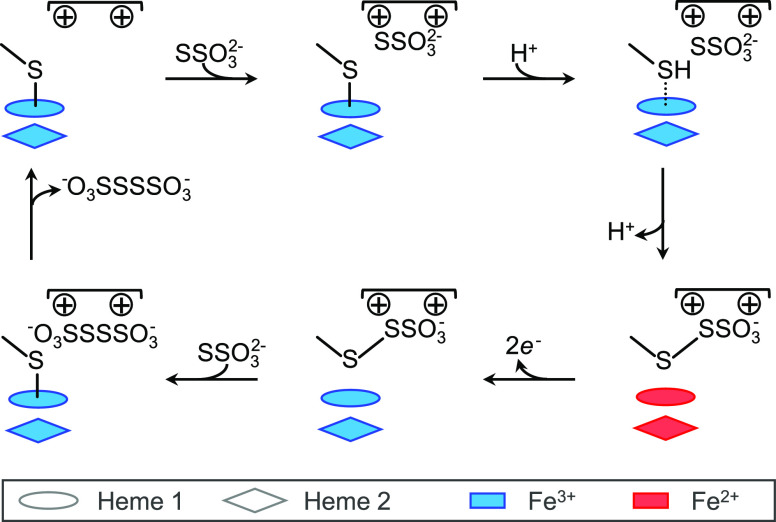
Proposed catalytic cycle for thiosulfate oxidation by TsdA enzymes where protonation of the active site cysteinate is critical to its dissociation from Heme 1. Two conserved Arg side chains in the substrate-binding pocket are indicated as positive charges.

With regard to the thiosulfate oxidizing activity of *Cj*TsdA, we anticipate that the binding site of thiosulfate overlaps with that of sulfite, such that thiosulfate would trigger similar protonation, dissociation, and subsequent reactivity of Cys^138^. Thus, we propose that this substrate triggered dissociation is essential to the mechanism of *Cj*TsdA thiosulfate oxidation, as illustrated in Figure 6. Binding of thiosulfate into the active site pocket raises the p*K*_a_ of Cys^138^, resulting in its protonation and subsequent dissociation from Heme 1. This raises the Heme 1 *E*_m_ from −134 to +75 mV such that together with Heme 2 (*E*_m_, +172 mV^31^), these sites can now accommodate the two electrons liberated during the oxidative conjugation of Cys^138^ and thiosulfate. We consider an alternative mechanism whereby thiosulfate reacts directly with the deprotonated Cys ligand to Heme 1 to be unlikely due to the low reduction potential^31^ of the His/Cys^–^-ligated Heme 1 that must accept an electron released in that process.

The product, Figure 6, of Cys^138^ conjugation by thiosulfate is cysteinyl thiosulfate (C_α_-CH_2_-S-SSO_3_^–^), a species that is widely accepted as an intermediate in the reaction of these enzymes^15,16,28^ and has direct analogy to the cysteinyl-sulfonate reported here. The midpoint potential for the oxidative addition of thiosulfate to cysteine has not been defined, but our results suggest a value of approximately 0 mV, which seems reasonable since it can be expected to lie between those of the thiosulfate/tetrathionate (+198 mV)^35^ and cysteine/cystine (−220 mV)^51^ couples. To complete the catalytic cycle for thiosulfate oxidation to tetrathionate (Figure 6), both hemes are then re-oxidized to their Fe(III) state by electron transfer to a suitable acceptor. Critically, the affinity of cysteinate for Fe(III)-heme can then provide the thermodynamic driving force for the thiol-disulfide exchange that forms tetrathionate and regenerates Cys^138^-ligated Heme 1. This sequence of events can explain why thiosulfonated di-Fe(II) *Av*TsdA remains unreactive toward a considerable molar excess of thiosulfate.^16^

The mechanism of Figure 6 illustrates how dissociation of a heme ligand reveals a redox center, Cys, that reacts with the substrate to exchange electrons with the nearby heme. This behavior is distinct to that in the other rare examples where heme ligand dissociation triggers catalytic activity by revealing a vacant heme site for substrate binding, for example, ref (13). The critical role for substrate-driven protonation to trigger Cys dissociation from the heme iron in *Cj*TsdA suggests that thiosulfate oxidation may proceed more rapidly at lower pH when protonation becomes increasingly favored. However, the steady-state catalytic rate of thiosulfate oxidation by *Cj*TsdA free in solution is dependent^33,37^ on the choice of the electron acceptor and pH such that the rate-limiting event(s) intrinsic to *Cj*TsdA have yet to be defined. With cytochrome *c* as an electron acceptor, *k*_cat_ decreases as the pH goes below 7 but the opposite is true with ferricyanide as an electron acceptor^37^ when *k*_cat_ is optimal at pH 4.0. However, these studies do allow us to place a lower limit on the rate of Cys^138^ conjugation by thiosulfate, as reported by the maximum turnover frequency (*k*_cat_) for thiosulfate oxidation. This value is approximately 800 s^–1^ at pH 6.5, 42 °C with cytochrome *c* as the electron acceptor^33^ and notably faster than the reaction with sulfite described here that was studied at pH 7 and 20 °C. Establishing the origin of this rate difference was beyond the scope of this study but could reasonably be expected to lie in different properties of the reactive, that is conjugating, S within sulfite and thiosulfate when these anions are bound in the enzyme-active site. For example the conjugating S may have a different position relative to S_γ_ of Cys^138^, charge density, and/or coordination geometry (trigonal pyramidal for sulfite and linear for thiosulfate).

Data available at the present time do not allow us to propose a mechanism for tetrathionate reduction by TsdA enzymes. However, a large activation barrier to Cys^138^ dissociation in the absence of tetrathionate, as found here for thiosulfate, has important implications. This offers a rationale for the observation that TsdA enzymes catalyze the interconversion of thiosulfate and tetrathionate in either direction when purified and assayed in vitro*.*^35,37^ TsdA activity in vivo^37^ is similarly modulated by the prevailing conditions such the directionality is likely to be determined by the *K*_d_ for binding of substrate(product) together with the cellular abundance of each.

Our mechanistic proposal for thiosulfate oxidation is consistent with and lends support to similar schemes proposed^16,17^ for both *Av*TsdA and SoxA based on modifications of active site cysteine residues that act as ligands to *c*-type heme in the enzymes as isolated. Purified SoxA has proven resistant to mechanistic investigation, whereas *Av*TsdA exhibits ligand switching and resultant complexity in Heme 2 redox properties that complicates a clean delineation of the active site (redox) chemistry. Nevertheless, we propose that the scheme illustrated in Figure 6 represents a common reaction pathway for both TsdA and SoxA enzymes where, in the case of the Sox proteins, the thiol disulfide exchange occurs with the cysteine of the partner protein SoxY, Reaction 1, rather than a second equivalent of thiosulfate, Reaction 2.

In closing, we consider the cysteine-ligated hemes of thiosulfate dehydrogenases in light of the recent classification^14^ of such centers. That ordering was based on spectroscopic attributes reporting different electronic structures which in turn underpin differences in reactivity and function. There is little correlation between the cysteine-ligated hemes of thiosulfate dehydrogenases and type-1 centers, for example, Figure S1A,C. While both systems require a dedicated electron-transfer chain for biological function, cysteine-heme ligation is not retained during the iron redox cycling associated with TsdA catalysis, instead the dissociated cysteine becomes a key redox center acting together with the heme to allow thiosulfate oxidation. The TsdA low-spin cysteinate-ligated ferric heme from which the cysteine can dissociate upon substrate-driven iron reduction is therefore reminiscent of the type-2 sites, Figure S1B. However, it is distinct in supporting redox catalysis in the dissociated state and with the substrate conjugating to the cysteine rather than ligating the heme iron. As such we propose an expansion of the original classification^14^ of thiolate-heme proteins to encompass the cysteine-ligated hemes exemplified by the active sites of TsdA and SoxA enzymes. A putative type-3 classification^14^ was proposed for centers with Cys/Cys ligation as found in the RNA-binding protein DGCR8.^52^ Thus, we propose a type-4 classification for the heme-thiolate centers that have evolved to catalyze the controlled redox transformation of inorganic oxo anions of sulfur, as described in this report. These type-4 centers are characterized by dissociation of the Cys-ligand in order to reveal both the thiol(ate) and heme as critical redox-active sites in oxidoreductases. The His/Cys^–^-ligated *c*-heme of DsrJ^53^ may also be a type-4 center. This heme exists in different ligation states, and sulfur-based chemistry of the Cys ligand is proposed as key to oxidation of a sulfur-containing substrate in the purple sulfur bacterium *A. vinosum*. The type-4 centers proposed here are associated with *c*-type heme, which is covalently bound to proteins via thioether linkages (Figure S1C). This provides a further distinction from the type-1 and -2 sites that are associated with *b*-type heme, which is attached to proteins only via the axial ligand(s) of the iron. However, we see no reason why *b*-hemes cannot provide type-4 centers in yet to be discovered enzymes.

## Supporting Information Available

Experimental methods, features of Cys-ligated hemes, sequence alignment, LCMS of *Cj*TsdA proteins, electronic absorbance spectra of *Cj*TsdA, and catalytic protein film voltammetry of *Cj*TsdA (PDF)

## Abbreviations

*Av*: *Allochromatium vinosum*
*Cj*: *Campylobacter jejuni*
*E*_m_: midpoint potential
LCMS: liquid chromatography-mass spectrometry
MCD: magnetic circular dichroism
TsdA: thiosulfate dehydrogenase
S.H.E: standard hydrogen electrode
SoxA: homologue of TsdA catalyzing the first step of the bacterial Sox thiosulfate oxidation pathway
