# Supplementary material for: Reaction of Thiosulfate Dehydrogenase with a Substrate Mimic Induces Dissociation of the Cysteine Heme Ligand Giving Insights into the Mechanism of Oxidative Catalysis
Source: J Am Chem Soc. 2022 Sep 29;144(40):18296–304. doi: 10.1021/jacs.2c06062 (PMC9562282; doi:10.1021/jacs.2c06062)
Supplement: Supplementary file 1 — ja2c06062_si_001.pdf [file ja2c06062_si_001.pdf]

Supporting Information.

Reaction of Thiosulfate Dehydrogenase with a Substrate Mimic Induces Dissociation of the Cysteine Heme Ligand Giving Insight into the Mechanism of Oxidative Catalysis.

Leon P. Jenner[1a], Jason C. Crack[1], Julia M. Kurth[2b], Zuzana Soldánová[1c], Linda Brandt[2], Katarzyna P. Sokol[3d], Erwin Reisner[3], Justin M. Bradley[1*], Christiane Dahl[2], Myles R. Cheesman[1*], Julea N. Butt[1*].

[1]Centre for Molecular and Structural Biochemistry, School of Chemistry and School of Biological Sciences, University of East Anglia, Norwich Research Park, Norwich, NR4 7TJ, United Kingdom.

[2]Institut für Mikrobiologie & Biotechnologie, Rheinische Friedrich Wilhelms Universität Bonn, 53115 Bonn, Germany.

[3]Yusuf Hamied Department of Chemistry, University of Cambridge, Lensfield Road, Cambridge CB2 1EW, United Kingdom.

* Julea N. Butt - Email: j.butt@uea.ac.uk

* Myles R. Cheesman - Email: m.cheesman@uea.ac.uk

* Justin M. Bradley - Email: justin.bradley@uea.ac.uk

Experimental Methods.

Reagents and buffers: Stocks (typically 100 mM) of sodium sulfite, potassium ferricyanide and sodium iodoacetate (Sigma-Aldrich) were prepared in 50 mM HEPES, 50 mM NaCl, pH 7 using deionized water (Resistivity = 18.2 MΩ cm, Milli-Q) within an anaerobic chamber (Belle Technologies) with a nitrogen atmosphere (<10 ppm O_2). Fresh stocks were prepared daily. Tris(2-carboxyethyl)phosphine hydrochloride (TCEP) was adjusted to pH 7 using ammonium hydroxide purchased as an aqueous 0.5 M solution sealed under argon gas (Sigma-Aldrich).

Protein purification: *C. jejuni* (*Cj*) TsdA was expressed in *E. coli* and purified as described previously.[1] The corresponding gene encodes the 309 amino acids predicted from C8J_0815 without the signal peptide, preceded by 15 N-terminal amino acids that include the Strep-II tag to facilitate purification. Residues are numbered from the first amino acid of this N-terminal extension.

Sample preparation: Samples were prepared in aqueous 50 mM HEPES, 50 mM NaCl, pH 7 buffer (with the exception of those used for nIR MCD measurement where D_2O replaced H_2O[2]) and transferred to an anaerobic chamber for incubation with anaerobic TCEP (final concentration, 1.5 mM), sulfite (final concentration, 1.5 mM) and/or ferricyanide (to achieve full re-oxidation) as indicated, Table S1. Samples (30 – 50 μM protein) for LC-MS were additionally treated with iodoacetate to acetylate free (per)thiols unless indicated otherwise. Excess sulfite or ferricyanide was then removed either by passage down a PD-10 desalting column (GE Healthcare) or by repeated buffer exchange in sealed 10 kDa MWCO spin concentrators (Thermo Fisher) using an AccuSpin micro 17 centrifuge (Thermo Fisher). Samples incubated anaerobically with sulfite in sealed cuvettes were monitored by electronic absorbance until there were no further spectroscopic changes (~60 minutes) prior to MCD measurements.

Table S1. Concentrations of *Cj*TsdA used in electronic absorbance and MCD measurements.

Figure	Sample	*Cj*TsdA concentration /μM
Fig. 2B Absorption	oxidized	136
	+ sulfite	66
	+ sulfite + ferricyanide	66
Fig. 4 MCD	oxidized (250 – 450 nm)	15
	oxidized (450 – 2000 nm)	130
	+ sulfite (1.5 mM) (250 – 700 nm)	34
	+ sulfite (1.5 mM) (700 – 2000 nm)	104
	+ sulfite + ferricyanide	46
	+ sulfite + ferricyanide	144
	+ sulfite + ferricyanide (700 – 2000 nm)	109

Electronic absorbance and magnetic circular dichroism (MCD): Absorption spectra were recorded using a model 4100 UV-visible-nIR spectrophotometer (Hitachi) or a model V-650 UV-visible spectrophotometer (JASCO). MCD spectra were recorded as described previously[3] with protein and buffer concentrations as shown in Table S1.

***Liquid Chromatography Mass Spectrometry (LC-MS)*:** Samples were diluted 10-fold with 2% acetonitrile (MS grade, Honeywell), 0.1% formic acid (Fluka) in water (MS grade, VWR), transferred to LC-MS vials containing 250 μL inserts (Agilent), and sealed within the anaerobic chamber before removal. A 20 μL aliquot of each

sample was loaded onto a ProSwift RP-1S column (4.6 x 50 mm) (Thermo Scientific) on an Ultimate 3000 uHPLC system (Dionex, Leeds UK). Bound proteins were eluted (0.2 mL min^{-1}) using a linear gradient over 15 min from 2% to 100% (v/v) acetonitrile, 0.1% (v/v) formic acid. The eluent was continuously infused into the electrospray ionization (ESI) source of a MicroTOF-QIII mass spectrometer, running Hystar (Bruker Daltonics, Coventry, UK) and operating in positive ion mode. The mass spectrometer was calibrated with ESI-L tuning mix (Agilent Technologies, California), which has m/z peaks in the range 118 – 2722. Compas Data Analysis 4.1, with Maximum Entropy v1.3, (Bruker) was used for processing of spectra under the LC peak, over mass range of 35 – 40 kDa. Mass spectrometry data are presented as fractional abundance.

To record LC-MS of protein studied by protein film electrochemistry (see below), the protein coated mesoporous hierarchically structured indium-tin oxide (ITO) working electrode was removed from the electrochemical cell and covered with a drop of iodoacetate solution (100 mM) for 2 min. The entire ITO layer was then scraped into a solution of 2% (v/v) acetonitrile, 0.1% (v/v) formic acid. The resulting suspension was then allowed to sediment by gravity (typically 5 min) so that the ITO particles settled. The protein containing solution was carefully recovered with a Hamilton syringe and analyzed as described above.

Protein Film Electrochemistry (PFE)*:** Non-catalytic PFE was recorded using mesoporous hierarchically structured indium-tin oxide working electrodes[4] (20 µm thickness, 0.25 cm^2 surface area, 750 nm pore diameter) on fluoride-doped tin oxide coated glass, using the procedure detailed previously[3] except as follows. After reagent addition and buffer exchange in spin concentrators as described in ***Sample preparation, samples were drop cast onto ice-cold electrodes. Subtraction of baseline electrode responses from cyclic voltammograms was performed using NOVA 1.11 software to leave just protein (Faradaic) responses. The latter were fit to the theoretical Nernstian response for a surface-adsorbed species:

$$i = \frac{n^2 F^2}{RT} \frac{\nu A_e \Gamma_O^* \exp\left(\left(\frac{nF}{RT}\right)(E - E_\mathrm{m})\right)}{\left(1 + \exp\left(\left(\frac{nF}{RT}\right)(E - E_\mathrm{m}\right)\right)^2}$$

where Γ_O^* is the surface population (mol cm^{-2}) of adsorbed redox active species and A_e is the electrode area (cm^2). R, F and T have their usual meanings and the number of electrons transferred in the half-reaction (n) is 1. Reported E_m values are averages obtained from the corresponding oxidative and reductive peak potentials.

Catalytic PFE, e.g. Fig. 6A,B, was recorded using TsdA-coated, rotating, pyrolytic graphite edge working electrodes using the procedure detailed previously.[1] Aliquots of sulfite, tetrathionate and thiosulfate were introduced as required to produce the desired concentration in the electrochemical cell.

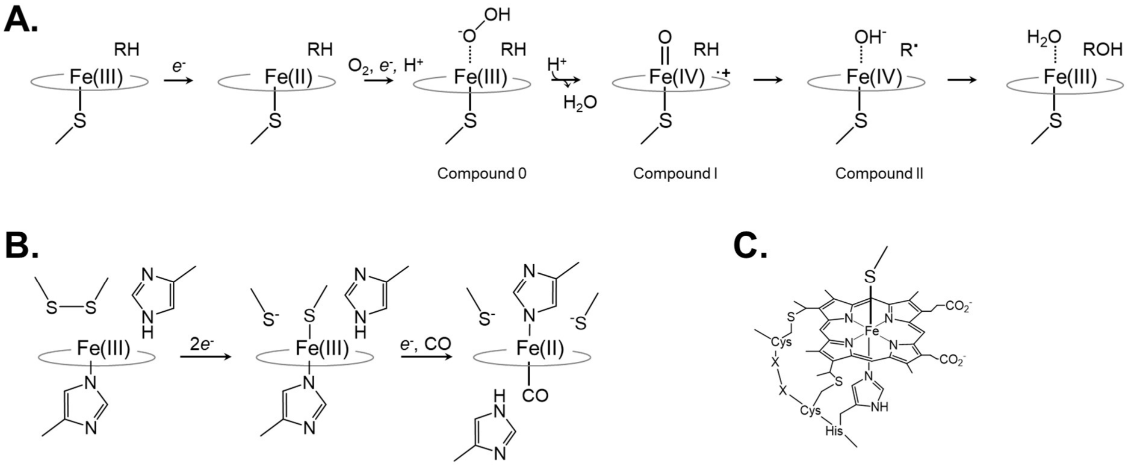

D.

```
Campylobacter jejuni        -------------GTIDETKMPKNSEYSKMVILGNKILNETSKYVGPQAKDPKKRFAGNN  77
Allochromatium vinosum      -----EEPPTV-ALTVPAAALLPDGALGESIVRGRRYLSDTPA--------QLPDFVGNG  46
Wolinella succinogenes      QYALPKFEGDVFWDTINAKPVVPDSIYGEFVQYGYELFVNTQAVIGPEVADPNMRYSGNN  95
Psychrobacter arcticus      --GTDTASGKP-TIKMPDESTIPDDEFGAAVRRGLQISNHTYK--------ELPNNVGNQ  95

Campylobacter jejuni        LSCSSCHANGG----SVQNQSGFVGIWARFPQYNARGDKVITLADRINGCFERSMNG---  130
Allochromatium vinosum      LACRHCHPGRDGEVGTEANAAPFVGVVGRFPQYSARHGRLITLEQRIGDCFERSLNG---  103
Wolinella succinogenes      LSCNSCHLGAG----TAKYAAPLVDNHANFPQYRNRENSLGTMAARVNGCMQRSMNG---  148
Psychrobacter arcticus      LNCTSCHLGNG----SEAYAAPWNNTPSVYPNYSKRTGRINTIQERINGCFERSLNG---  148

Campylobacter jejuni        ---KRMPSDTPEMKAMLTYMQWLSQGVPVGA-KIEGQGLKKIDF---ISRAADPKKGKAI  183
Allochromatium vinosum      ---RALALDHPALIDMLAYMSWLSQGVPVGA-VVAGHGIPTLTL---E-REPDGVHGEAL  155
Wolinella succinogenes      ---YPLPAEGKEMKAFLAYIHWLGQGIPVGA-KIEGRSLKTVDRKMVQQNAADVKNGAEV  204
Psychrobacter arcticus      ---KALDLNSDDMNAMVSYMSWLSQDMPFGV-SPEGSGFVKVDK---T-LEPNTDNGKKL  200

Campylobacter jejuni        YMDKCAVCHQENGLGLKNEDSA--GAYYLYPPLWGD-DSYNTGAGMYRLIKAASYIKENM  240
Allochromatium vinosum      YQARCLACHGADGSGTLDAD-----GRYLFPPLWGP-RSFNTGAGMNRQATAAGFIKHKM  209
Wolinella succinogenes      YARDCASCHGAEGEGLRRESKDGKPAGYEFPPLWGSDDTYNTGAGMYRTLKAADFIKSTM  264
Psychrobacter arcticus      FAEKCSVCHGATGEGQYNDD-----GTYVYPAIAGD-KSFNDGAGMARTYTAASFIKGKM  254

Campylobacter jejuni        PQGAP-DLSLEDAYDVAAYMNSQ--ARPIKANRDKDFPDRKVK---PLDMD---VGPYDD  291
Allochromatium vinosum      PLGADDSLSDEEAWDVAGFVLTH--PRPLFQEPTGD------------------------  243
Wolinella succinogenes      PKGAP-TLSDKDAYDVAAFINDYSHYRTVKLNRQNDFVDPKVR---VPDHDQ--PGPYGP  318
Psychrobacter arcticus      PFGQGGSLSDQEAVDIASYFTHL--PRPIKANKDKDWPNGDAPKDVRR------------  300

Campylobacter jejuni        S---------FSTTQHRYGPYTNMIKK-------  309
Allochromatium vinosum      ----------------------------------  243
Wolinella succinogenes      EGSYIFPDEGKTQMDYKVGPYKGIIKQKPAAK--  350
Psychrobacter arcticus      ----------------------------------  300
```

Figure S1.

A) Cys$^-$ ligation of *b*-heme is retained by type-1 sites during the redox cycling associated with catalysis as illustrated here for cytochromes P450.

B) Transitory Cys$^-$ ligation of *b*-heme is typical of type-2 sites for sensing heme and gas molecules.

C) Cys$^-$/His ligated *c*-heme.

D) Sequence alignment for TsdAs from the indicated organisms. Heme 1 binding motif, proximal His ligand and distal Cys ligand are red on pink. Heme 2 binding motif, proximal His ligand and distal Met ligand are red on gray. Arg residues in the catalytic pocket of *A. vinosum* TsdA[5] are black on yellow.

RESULTS

LC-MS of CjTsdA proteins: To date, all LC-MS measured[6] for unmodified forms of *Cj*TsdA expressed in *Escherichia coli* reports an additional mass of 99 (± 2) Da compared to that anticipated on the basis of the protein sequence with the addition of two hemes, an N-terminal Step II tag and four linker residues.[7] This is readily illustrated by the LC-MS for *Cj*TsdA C138H (Fig. S2A) and *Cj*TsdA C138M (Fig. S2B), which lack Cys[138] as a site of covalent modification. Both variants exhibit no significant heterogeneity and the observed (*anticipated from plasmid sequence*) masses are 37 240 (*37 137*) and 37 230 (*37 131*) Da for the C138M and C138H variants respectively. Experiments to date have unfortunately failed to identify the source of the additional mass of the protein but others have reported +98 adducts due to sulfate and/or phosphate bound *non-covalently* to peptides and proteins.[8] Accounting for this additional mass of the protein allows for straightforward interpretation of the LC-MS displayed by *Cj*TsdA with cysteine modifications as shown in Fig. S3. Anticipated masses are shown in Fig. S4 for forms of TsdA that contain a modified Cys[138].

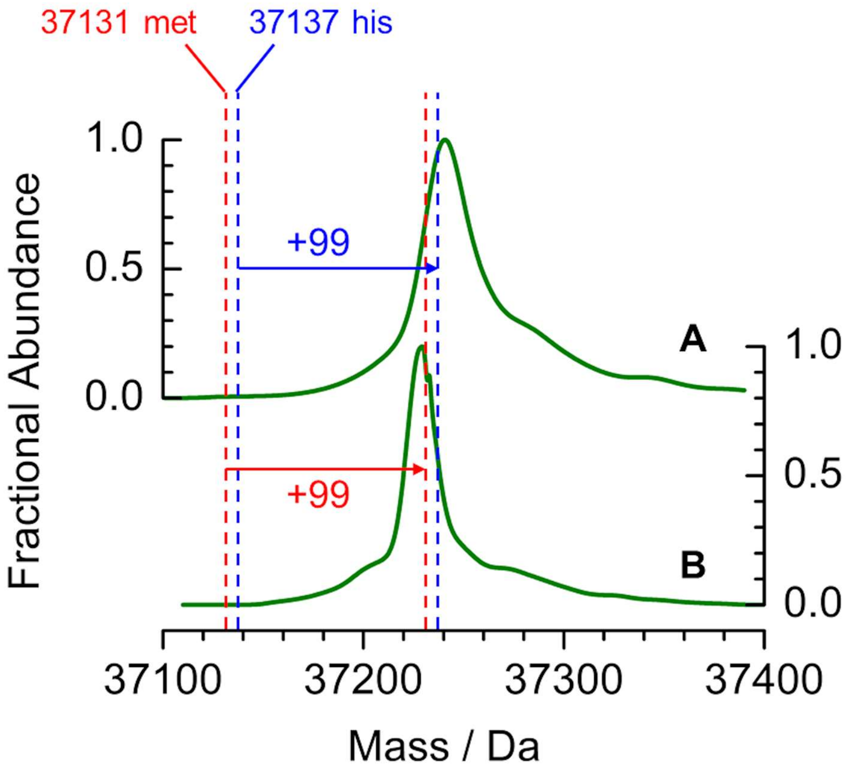

Figure S2. Deconvoluted mass spectra of *Cj*TsdA proteins. (A) as prepared C138H *Cj*TsdA. **(B)** as prepared C138M *Cj*TsdA. For each variant, the vertical dashed lines are located at the anticipated masses and at those masses +99 Da.

The LC-MS of as-prepared *Cj*TsdA, Fig. S3A, without treatment with iodoacetate shows three-fold heterogeneity. One major species is observed near the mass associated with protein containing unmodified Cys[138]. A second, similarly abundant form, has a mass corresponding to the addition of either a single sulfur atom or two oxygen atoms, consistent respectively with persulfuration or sulfinylation of cysteine. A lack of high-spin species in electronic spectra of as-prepared TsdA[3] suggests that a sulfinylated form is not present in solution but does not rule out the possibility that oxidative modification occurs during handling and preparation of samples for LC-MS. A third, minor, species suggests a small population of cysteinyl thiosulfate.

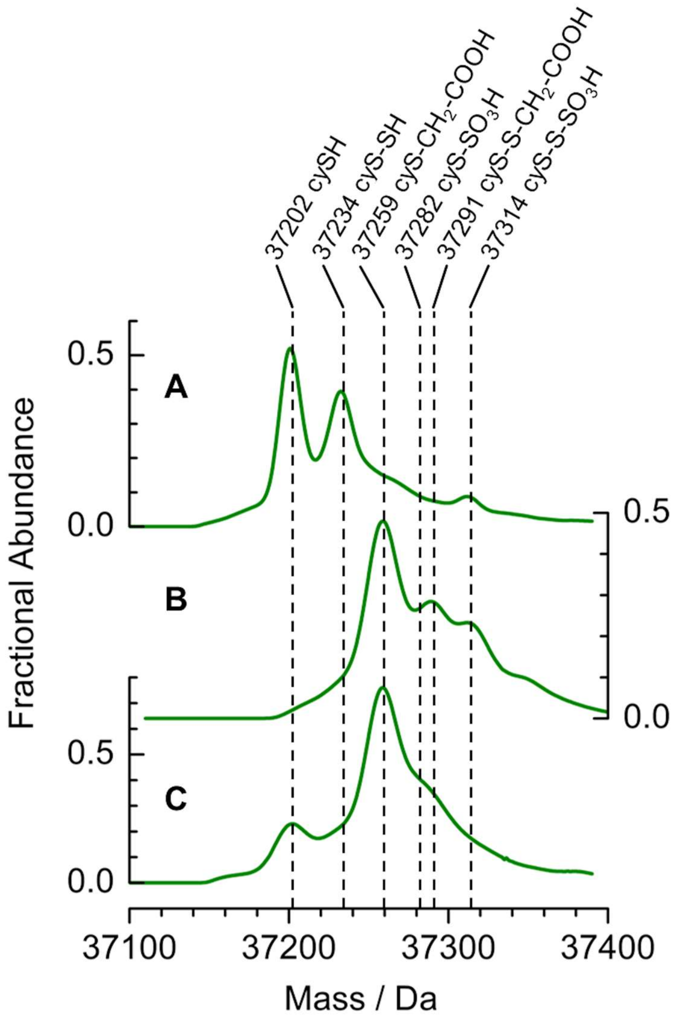

Figure S3. Deconvoluted mass spectra of *Cj*TsdA proteins. (A) as prepared *Cj*TsdA. **(B)** as prepared *Cj*TsdA treated with iodoacetate prior to LC-MS. **(C)** as prepared *Cj*TsdA incubated with TCEP and then iodoacetate prior to LC-MS. See methods for details.

Persulfurated and unmodified cysteine forms will differ from sulfinylated Cys^{138} in that both will react with the alkylating agent iodoacetic acid and in the process be protected from adventitious oxidative modifications prior to LC-MS analysis. The LC-MS of TsdA treated with iodoacetic acid is shown in Fig. S3B. The major peak corresponds to the +57 Da shift that would result from alkylation of Cys^{138}. Two smaller features lie at masses associated with alkylated persulfurated and cysteinyl thiosulfate. The absence of a feature near 37 234 Da implies that, although it contained the persulfurated form, there was no sulfinylated Cys^{138} in the as-prepared TsdA.

To produce homogeneous Cys^{138}-unmodified *Cj*TsdA for the sulfite incubation experiments presented in the main text, samples were pre-treated with the disulfide reducing agent tris(2-carboxyethyl)phosphine[9-10] (TCEP). After TCEP treatment and subsequent trapping with iodoacetate the LC-MS, Fig. S3C and Fig. 2A, is dominated the acetylated cysteine form with some unreacted cysteine. TCEP treatment does therefore yield a homogeneous material with unmodified Cys^{138}.

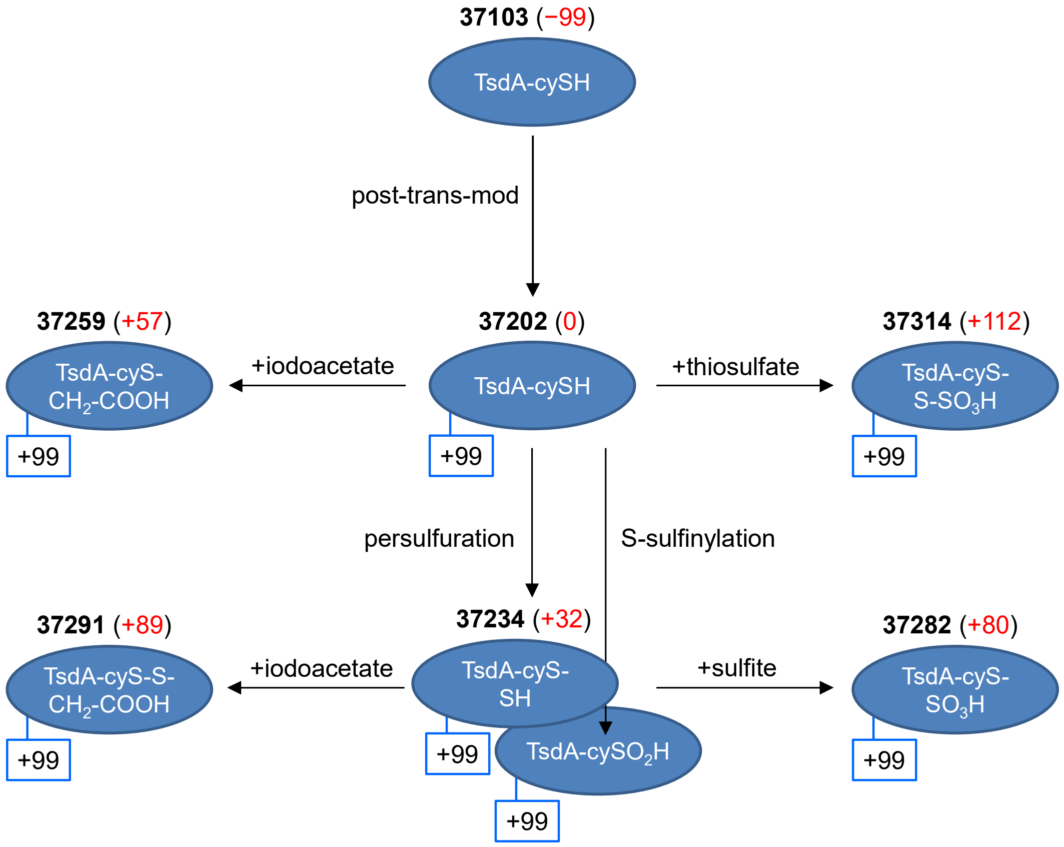

Figure S4. Predicted masses (Da) of *Cj*TsdA. The bracketed numbers in red show the mass changes for each species relative to the mass of *Cj*TsdA *with* the post-translational (+99 Da) modification and an unmodified Cys^{138}. All masses include the two covalently bound hemes. The nature of the +99 Da modification was not identified in this study but others have reported +98 adducts due to sulfate and/or phosphate bound non-covalently to peptides and proteins.[8]

Low-spin Hemes in* Cj*TsdA reduced by ascorbate or dithionite: The products of *Cj*TsdA reduction by ascorbate and dithionite contain only low-spin heme as revealed by electronic absorbance spectroscopy, Fig. S5, and MCD.[3]

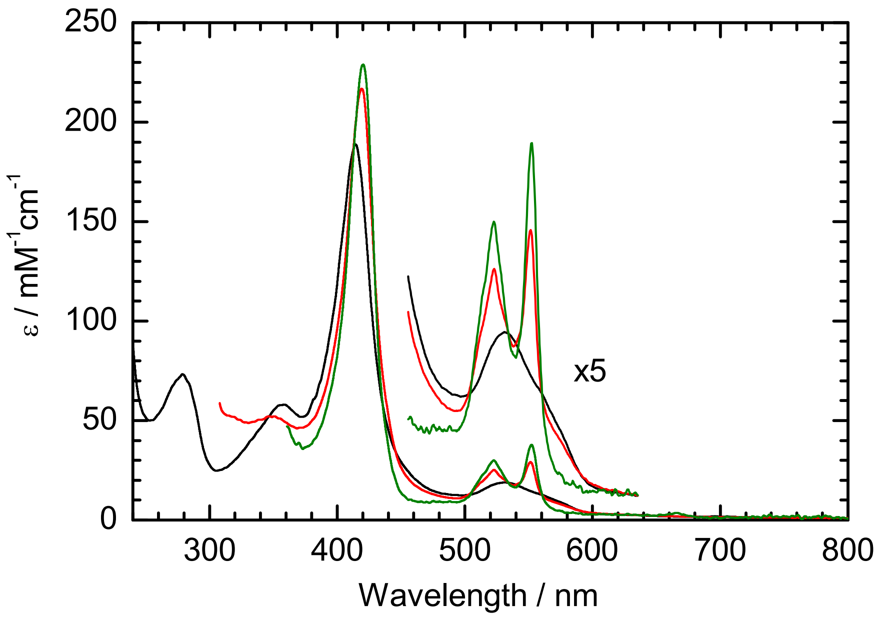

Figure S5. Properties of oxidized di-Fe(III) *Cj*TsdA (black), and after anaerobic incubation with 1.5 mM sodium ascorbate (red), then 1.5 mM sodium dithionite (green). Samples in anaerobic 50 mM HEPES, 50 mM NaCl, pH 7.

Steady-State Analysis of* Cj*TsdA Catalytic Activity and its Inhibition by Sulfite. Quantitative insight into the catalytic activity of *Cj*TsdA was provided by PFE. The thiosulfate oxidase activity of *Cj*TsdA was quantified by cyclic voltammetry through positively signed catalytic waves above approximately 0.1 V *vs* SHE, e.g. Fig. S6A. Cyclic voltammetry in the presence of tetrathionate revealed negative catalytic currents due to substrate reduction below approximately 0.25 V *vs* SHE, e.g. Fig. S6B. Addition of sulfite resulted in inhibition of oxidative and reductive catalysis as indicated by the corresponding drop in catalytic current magnitudes, Fig. S6 A and B respectively.

Insight into the mechanism of sulfite inhibition was obtained from experiments in which steady-state catalytic currents were recorded as tetrathionate (thiosulfate) was introduced to a solution of defined sulfite concentration. For both substrates at each sulfite concentration the catalytic current magnitude varied with substrate concentration in the manner predicted by the Michaelis-Menten equation, Fig. S6 C and D. The corresponding Lineweaver-Burk plots revealed sulfite as a competitive inhibitor of both oxidative and reductive catalysis, Fig. S6 C and D, with K_M increased and V_{max} unchanged with increased sulfite concentration. The results illustrate rapid inhibition of TsdA by sulfite during the conditions of these experiments where measurements are complete in a few minutes and the hemes are being continually redox cycled. Thus, they may represent simple competitive (steric) inhibition of substrate binding in the active site pocket. However, additional complexity in the inhibition of *Cj*TsdA by sulfite was revealed during chronoamperometry (i.e. measurement of current at fixed potential) that revealed increased inhibition over several minutes that is most likely a consequence of slow formation of sulfonated cysteinyl[138]. Discussion of that data is beyond the scope of this report.

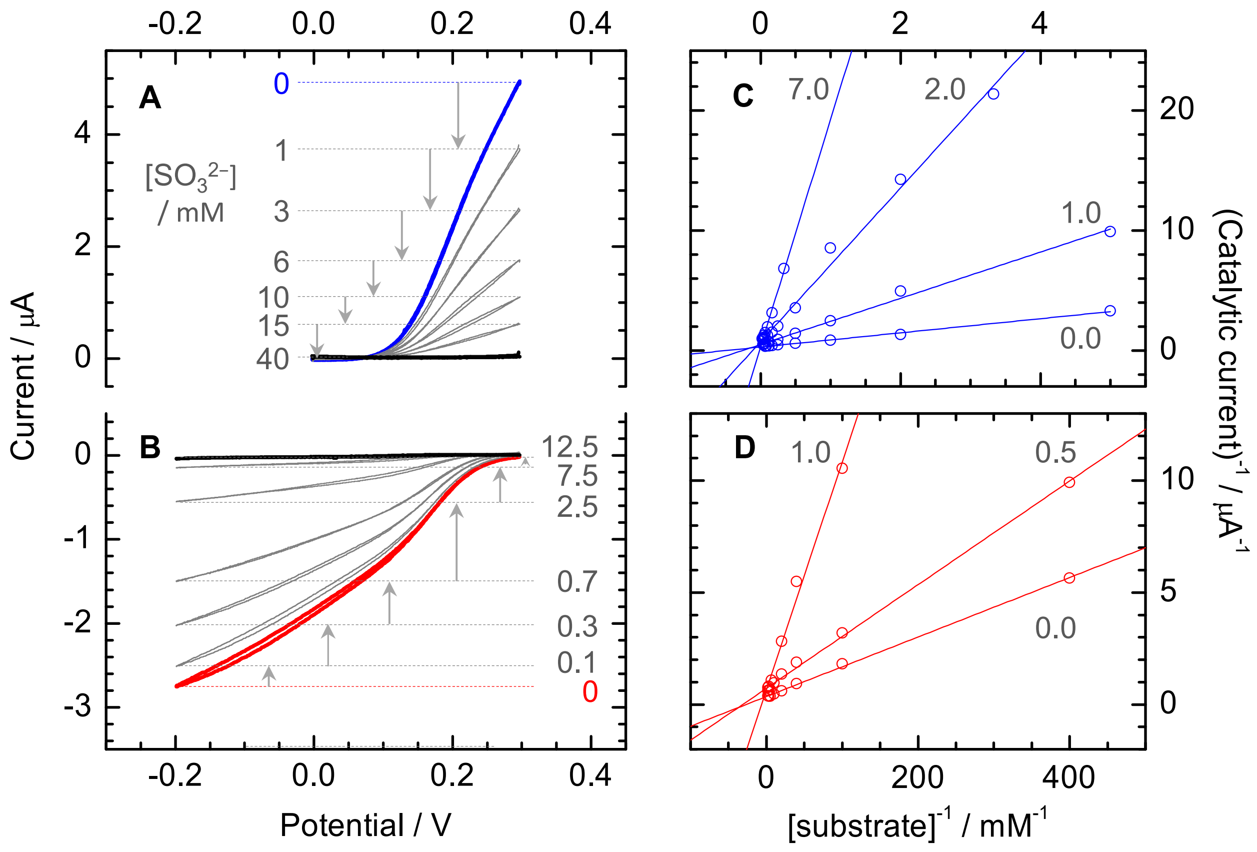

Figure S6. Sulfite inhibition of thiosulfate oxidation (top) and tetrathionate reduction (bottom) by TsdA.

Baseline subtracted cyclic voltammograms of *Cj*TsdA adsorbed on PGE electrodes in the presence of **(A)** 8 mM thiosulfate (blue line) or **(B)** 0.3 mM tetrathionate (red line). For each, the concentrations of sulfite are shown in gray. Lineweaver-Burk plots showing the catalytic current at the indicated sulfite concentrations for **(C)** thiosulfate oxidation and **(D)** tetrathionate reduction (symbols = data, lines = linear fit). Measurements were made in 100 mM ammonium acetate, 50 mM NaCl, pH 5. Electrode rotation at 500 rpm, scan rate 10 mV s^{-1} and T = 315 K.

References.

(1) Kurth, J. M.; Dahl, C.; Butt, J. N. Catalytic Protein Film Electrochemistry Provides a Direct Measure of the Tetrathionate/Thiosulfate Reduction Potential. *J. Am. Chem. Soc.* **2015,** *137*, 13232-13235.
(2) Cheesman, M. R.; Greenwood, C.; Thomson, A. J. Magnetic Circular Dichroism of Hemoproteins. *Adv. Inorg. Chem.* **1991,** *36*, 201-255.
(3) Jenner, L. P.; Kurth, J. M.; van Helmont, S.; Sokol, K. P.; Reisner, E.; Dahl, C.; Bradley, J. M.; Butt, J. N.; Cheesman, M. R. Heme Ligation and Redox Chemistry in Two Bacterial Thiosulfate Dehydrogenase (TsdA) Enzymes. *J. Biol. Chem.* **2019,** *294*, 18002-18014.
(4) Mersch, D.; Lee, C. Y.; Zhang, J. Z.; Brinkert, K.; Fontecilla-Camps, J. C.; Rutherford, A. W.; Reisner, E. Wiring of Photosystem II to Hydrogenase for Photoelectrochemical Water Splitting. *J. Am. Chem. Soc.* **2015,** *137*, 8541-8549.
(5) Grabarczyk, D. B.; Chappell, P. E.; Eisel, B.; Johnson, S.; Lea, S. M.; Berks, B. C. Mechanism of Thiosulfate Oxidation in the SoxA Family of Cysteine-Ligated Cytochromes. *J. Biol. Chem.* **2015,** *290*, 9209-9221.
(6) Jenner, L. P. Spectroscopic and Electrochemical Studies of Di-Heme Thiosulphate Dehydrogenases. University of East Anglia, Norwich, UK, 2019.
(7) Kurth, J. M.; Butt, J. N.; Kelly, D. J.; Dahl, C. Influence of Haem Environment on the Catalytic Properties of the Tetrathionate Reductase TsdA from *Campylobacter jejuni*. *Biosci. Rep.* **2016,** *36*, e00422.
(8) Chowdhury, S. K.; Katta, V.; Beavis, R. C.; Chait, B. T. Origin and Removal of Adducts (Molecular Mass = 98 U) Attached to Peptide and Protein Ions in Electrospray Ionization Mass-Spectra. *J. Am. Soc. Mass Spectrom.* **1990,** *1*, 382-388.
(9) Burns, J. A.; Butler, J. C.; Moran, J.; Whitesides, G. M. Selective Reduction of Disulfides by Tris(2-Carboxyethyl)Phosphine. *J. Org. Chem.* **1991,** *56*, 2648-2650.
(10) Getz, E. B.; Xiao, M.; Chakrabarty, T.; Cooke, R.; Selvin, P. R. A Comparison Between the Sulfhydryl Reductants Tris(2-carboxyethyl)phosphine and Dithiothreitol for Use in Protein Biochemistry. *Anal. Biochem.* **1999,** *273*, 73-80.
